# Glucose and Inositol Transporters, SLC5A1 and SLC5A3, in Glioblastoma Cell Migration

**DOI:** 10.3390/cancers14235794

**Published:** 2022-11-24

**Authors:** Philippa K. Brosch, Tessa Korsa, Danush Taban, Patrick Eiring, Philipp Kreisz, Sascha Hildebrand, Julia Neubauer, Heiko Zimmermann, Markus Sauer, Ryo Shirakashi, Cholpon S. Djuzenova, Dmitri Sisario, Vladimir L. Sukhorukov

**Affiliations:** 1 Department of Biotechnology & Biophysics, Biocenter, University of Würzburg, 97074 Würzburg, Germany; philippa.brosch@uni-wuerzburg.de (P.K.B.); tessa.korsa@ibmt.fraunhofer.de (T.K.); danush.taban@uni-wuerzburg.de (D.T.); patrick.eiring@uni-wuerzburg.de (P.E.); sascha.hildebrand@stud-mail.uni-wuerzburg.de (S.H.); m.sauer@uni-wuerzburg.de (M.S.); 2 Fraunhofer Institute for Biomedical Engineering (IBMT), 66280 Sulzbach, Germany; julia.neubauer@ibmt.fraunhofer.de (J.N.); heiko.zimmermann@ibmt.fraunhofer.de (H.Z.); 3 Julius-von-Sachs Institute, University of Würzburg, 97082 Würzburg, Germany; philipp.kreisz@uni-wuerzburg.de; 4 Department of Molecular and Cellular Biotechnology, Saarland University, 66123 Saarbrücken, Germany; 5 Faculty of Marine Science, Universidad Católica del Norte, Coquimbo 1281, Chile; 6 Institute of Industrial Science, The University of Tokyo, Tokyo 153-8505, Japan; aa21150@iis.u-tokyo.ac.jp; 7 Department of Radiation Oncology, University Hospital of Würzburg, 97080 Würzburg, Germany; djuzenova_t@ukw.de

**Keywords:** volume regulation, transportome, phlorizin

## Abstract

**Simple Summary:**

Cell migration is the main obstacle to the treatment of highly invasive brain cancer glioblastoma multiforme (GBM). We investigated in vitro the potential role of two solute carrier proteins (SLCs), SLC5A1 and SLC5A3, and their respective substrates, glucose and inositol, in GBM cell migration. We found that GBM cell motility was increased by medium supplementation with glucose and inositol and was strongly impaired by inhibition of SLC5A1/3 proteins. Using conventional and super-resolution fluorescence microscopy, we showed that both SLCs were not only highly expressed in migrating GBM cells, but they also localized to the lamellipodia, i.e., the migration-governing cell protrusions. Taken together, our data suggest that SLC5A1 and SLC5A3 are involved in GBM cell migration, presumably by mediating solute transport, osmotic water fluxes and thus local volume regulation in the lamellipodium.

**Abstract:**

(1) Background: The recurrence of glioblastoma multiforme (GBM) is mainly due to invasion of the surrounding brain tissue, where organic solutes, including glucose and inositol, are abundant. Invasive cell migration has been linked to the aberrant expression of transmembrane solute-linked carriers (SLC). Here, we explore the role of glucose (SLC5A1) and inositol transporters (SLC5A3) in GBM cell migration. (2) Methods: Using immunofluorescence microscopy, we visualized the subcellular localization of SLC5A1 and SLC5A3 in two highly motile human GBM cell lines. We also employed wound-healing assays to examine the effect of SLC inhibition on GBM cell migration and examined the chemotactic potential of inositol. (3) Results: While GBM cell migration was significantly increased by extracellular inositol and glucose, it was strongly impaired by SLC transporter inhibition. In the GBM cell monolayers, both SLCs were exclusively detected in the migrating cells at the monolayer edge. In single GBM cells, both transporters were primarily localized at the leading edge of the lamellipodium. Interestingly, in GBM cells migrating via blebbing, SLC5A1 and SLC5A3 were predominantly detected in nascent and mature blebs, respectively. (4) Conclusion: We provide several lines of evidence for the involvement of SLC5A1 and SLC5A3 in GBM cell migration, thereby complementing the migration-associated transportome. Our findings suggest that SLC inhibition is a promising approach to GBM treatment.

## 1. Introduction

Glioblastoma multiforme (GBM) is a high-grade astrocytoma with a dismal prognosis. Even after surgical resection and radiochemotherapy, the median survival time for patients does not exceed 15 months [1,2]. Recurrence is mainly due to the diffuse invasion of tumor cells into the surrounding brain tissue [3,4]. Thus, considerable efforts have been made to inhibit GBM cell migration [5,6,7], with many studies focusing on cytoskeletal dynamics at the leading edge of lamellipodium [8,9,10,11,12].

In addition to lamellipodia and ruffles [13], the leading edge of migrating cancer cells frequently displays transient spherical protrusions of the plasma membrane, known as “blebs” [5,14]. Preceding bleb nucleation, the actin cortex underlying the membrane becomes locally ruptured or detached, allowing the formation of membrane protrusion. Blebs, initially devoid of filamentous actin, then rapidly inflate and eventually, upon reconstruction of the actin cortex, contract and disappear [15,16].

According to the osmotic engine migration model [17], a variety of membrane transporters and channels enable a net water/solute-exchange at the anterior and posterior end of the cell. Interestingly, an increasing number of studies on highly motile cancer cells have reported a polarized distribution of water and ion channels, collectively known as the migration-associated transportome [17,18].

In addition to inorganic ions, various small organic osmolytes (SOOs) are also involved in the cell volume regulation of mammalian cells [19]. Among the most abundant SOOs in the human brain are glucose [20,21] and inositol [22,23,24,25]. Aberrant inositol levels have been linked to glial proliferation, high-grade astrocytoma and glioblastoma multiforme [26,27], and the increased glucose uptake of cancer cells has been studied extensively [28].

Cellular glucose and inositol uptake occur via specific transporters belonging to the solute carrier (SLC) 5-gene family [29,30]. SLC5A1, also known as sodium/glucose cotransporter 1 (SGLT1), mediates the cellular uptake of glucose, whereas inositol transport through cell membranes is achieved by SLC5A3, also known as sodium/myo-inositol cotransporter (SMIT1). Increasing evidence points to the involvement of SLCs in cancer progression. Not only are many SLC-transporters upregulated in tumor cells [31,32], but recently, they have come into focus as a marker of cancer phenotype [33,34]. Interestingly, both SLC5A1 and SLC5A3 are highly expressed in various types of cancer [35].

While glucose is the main metabolic substrate required for cancer cell survival and proliferation [36], inositol serves as an important osmolyte in cell volume regulation and fluid homeostasis, particularly in the brain [37]. Inositol is also the precursor of phosphatidylinositol 4,5-bisphosphate (PIP2), known as a key signaling lipid in cancer cell migration [38].

However, little is known about the subcellular expression of the glucose and inositol transporters SLC5A1 and SLC5A3 and their possible involvement in GBM cell migration. In this study, we thus examined the impact of glucose and inositol, as well as SLC inhibition, on the migration of two highly motile glioblastoma cell lines, DK-MG and SNB19. We also visualized, using immunostaining and fluorescence microscopy, the intracellular localization of both transporters in migrating and non-motile GBM cells.

## 2. Materials and Methods

### 2.1. Cell Culture

Both human glioblastoma (GBM) cell lines, DK-MG and SNB19, were acquired from DSMZ (Braunschweig, Germany) and routinely cultured under standard conditions (5% CO_2_, 37 °C) in complete growth medium (CGM), consisting of Dulbecco’s modified Eagle’s medium (DMEM, Sigma, Deisenhofen, Germany) supplemented with 10% FBS. Cells were used at low (<15) passages after thawing. In addition, they were authenticated on the basis of morphology, expression of PTEN and p53, and growth curve analysis and were regularly tested for Mycoplasma (MycoAlert; Lonza, Rockland, ME, USA).

### 2.2. Immunostaining

DK-MG or SNB19 cells were seeded at either 5 × 10^4^ or 5 × 10^3^ cells per well, respectively, on eight-well chambered cover glasses (Sarstaedt, Nümbrecht, Germany) and then washed in pre-warmed (37 °C) phosphate-buffered saline (PBS). Cells were fixed using a PBS solution containing 0.25% glutaraldehyde and 4% paraformaldehyde (methanol-free) for 10 min at room temperature. Cells were then washed thrice in PBS for 5 min. Permeabilization was performed with 0.1% Triton X-100 in PBS for 10 min, followed by three washing steps with PBS for 5 min. Thereafter, the samples were incubated with blocking buffer containing 5% bovine serum albumin (BSA; Sigma Aldrich, St. Louis, MO, USA) in PBS for one hour. Following the blocking phase, cells were incubated for one hour with either rabbit SMIT1 antibodies (Abcam, Cambridge, UK) or goat SGLT1 antibodies (Everest Biotech, Bicester, UK) diluted 1:400 in blocking buffer. After incubation with primary antibodies, the samples were washed 3 times in PBS for 5 min. The cells were stained by incubating them in blocking buffer with 1:200-diluted Alexa Fluor (AF) 532-conjugated goat anti-rabbit IgG (Thermo Fisher Scientific, Waltham, MA, USA), AF488-conjugated rabbit anti-goat IgG (Thermo Fisher Scientific, Waltham, MA, USA), AF647 AffiniPure F(ab’)_2_ fragment donkey anti-rabbit IgG (Jackson ImmunoResearch, Ely, UK) or AF647-conjugated donkey anti-goat IgG (Invitrogen, Waltham, MA, USA) for 1 h, respectively. Finally, the cells were washed 3 times with PBS. For actin-staining experiments, cells were additionally incubated overnight with phalloidin-conjugated AF647 or AF532 (Thermo Fisher Scientific, Waltham, MA, USA) diluted 1:200 in PBS at 4 °C and washed with PBS prior to measurement. For microtubule staining, following SLC staining, the cells were incubated for 1 h in mouse anti-β-tubulin antibodies (Sigma Aldrich, St. Louis, MO, USA) diluted 1:200 in blocking buffer.

Immunostaining of wounded cell monolayers was performed by seeding 5 × 10^5^ (DK-MG) or 2 × 10^5^ (SNB19) cells per well on eight-well chambered cover glasses and incubated under standard conditions. Upon reaching confluence, the monolayer was scratched with a pipette tip and the culture medium was replaced to remove the detached cell debris. The cells were then incubated for 3 h under standard conditions until staining was performed, as described above.

### 2.3. Cell Volumetry

Volumetric measurements were performed to analyze cell volume regulation upon exposure to inositol, glucose, mannitol and sucrose, as described previously. [19,39] Briefly, detached cells were placed in an observation chamber and allowed to adhere to a poly-D-lysine-coated glass slide for 10 min prior to observation. Images were taken at 10-s intervals for 20 min. During measurement, the medium was replaced using a syringe pump with a perfusion speed set to 20 µL/s. Cell volume changes were induced by applying SOO solutions of 150 mOsm. Perfusion media (pH 7.4) contained 4.6 mOsm of K_2_HPO_4_/KH_2_PO_4_, 0.1 mM CaAc_2_ and 0.02 mM MgAc_2_, yielding ~5 mOsm of inorganic electrolytes. Each experiment was carried out at least 3 times. Starting 30 s into video recording, 0.5 mL SOO solution was pumped through the chamber. Frames 1–3 served as a reference for isotonic cell volume (V_0_). Cell volume was determined by assuming spherical geometry and measuring the cross-section area with a custom-made ImageJ plugin [19]. From these volumetric data, we used the following equation to calculate the SOO-dependent regulatory volume decrease (RVD) inhibition index [40]:

(1) $$IC_{RVD}=\left(\frac{v_{20}-v_{0}}{v_{5}-v_{0}}\right)*100\%$$

with $\nu_{0}=1$ as normalized isotonic cell volume and $\nu_{5}$ and $\nu_{20}$ as the normalized cell volumes after 5- and 20-min exposure to hypotonic medium, respectively. As pointed out elsewhere [40], an *IC_RVD_* ≈ 0 means complete recovery of initial cell volume, while *IC_RVD_* = 100% shows that RVD was abolished and the cells remained swollen. An *IC_RVD_* larger than 100% indicates continuous secondary swelling.

### 2.4. Confocal Laser Scanning Microscopy (CLSM)

Confocal fluorescence images were acquired with a Zeiss confocal LSM 700 microscope using a Plan-Apochromat 63×/1.40 oil immersion objective (Zeiss, Jena, Germany) and argon laser light excitations at 488, 555 and 639 nm. Image processing was carried out with ImageJ software.

### 2.5. dSTORM (Direct Stochastic Optical Reconstruction Microscopy)

Reversible photoswitching of the dyes AlexaFluor 647 and AlexaFluor 532 was performed in a photoswitching buffer containing 100 mM mercaptoethylamine (Sigma) in PBS at pH ~7.4. A detailed experimental setup for *d*STORM was described previously [41]. Measurements were performed in 8-well chambered cover glasses (Sarstaedt, Nümbrecht, Germany). For localization data processing and image reconstruction, the open access software *rapid*STORM 3.2 was used, as previously described [42,43]. Image processing was performed with ImageJ. Localization densities were determined via an in-house developed Python-based DBSCAN (“Density Based Spatial Clustering of Applications with Noise”) algorithm.

### 2.6. Gene Expression Profiling Interactive Analysis of SLC5A1 and SLC5A3 in GBM

The bioinformatics tool Gene Expression Profiling Interactive Analysis (GEPIA2 [44], http://gepia.cancer-pku.cn/index.html (accessed on 24 February 2021)) was used to analyze patient survival data and tumor-vs.-normal differential expression data from the freely accessible data banks Genotype-Tissue Expression project (GTEx) and The Cancer Genome Atlas (TCGA) regarding SLC5A1- and SLC5A3-expression in GBM cells.

### 2.7. Wound Healing Assay

Cell migration rates were analyzed via a scratch assay. One day prior to measurements, 4 × 10^5^ cells/mL were seeded in a Petri dish (diameter 35 mm) in 2 mL of CGM supplemented with 30 mM glucose (GLU) or inositol (INO). CGM supplemented with 30 mM of metabolically inert mannitol (MAN) served as a tonicity control. Before starting the video microscopy experiments, several wounds were introduced into the confluent cell monolayer by gentle scratching with a pipette tip, followed by removal of culture media, a washing step with PBS and addition of the respective SOO-supplemented media. Afterwards, the cells were placed for 18 h in an incubator (37 °C, 5% CO_2_, 20% O_2_) with an integrated camera (Biostation IM-Q, Nikon, Melville, NY, USA). For the inhibition experiments, 50 nM of phlorizin was present 3 h prior to and during the video recording. Phlorizin is a competitive inhibitor of SLC5A1 [45] and inhibits SLC5A3 [46]. For each experimental condition, ~10 regions of interest (ROI) were recorded for 18 h. Images of each ROI were acquired every 10 min with an image resolution of 1600 × 1200 and a pixel size of 0.44 µm^2^/pixel. The wound closure rate (µm^2^/min) for each condition was then determined as previously described [5,7].

### 2.8. Statistical Analysis

Data are presented as mean ± SE unless otherwise noted. A Student’s unpaired *t*-test was performed when statistical comparisons were made between two sets of data. A *p*-Value of <0.05 was considered significant and indicated by “*” where applicable.

## 3. Results

Increasingly, aberrant expression of the SLC5 sub-family members has been linked to cancer cell invasion and metastasis [33]. Biopsy analysis of GBM also revealed expression of the proteins SLC5A1 and SLC5A3 in brain tumors (Supplementary Materials Figure S1A,B). Moreover, glucose and *myo*-inositol, the substrates of these transporters, are abundant in brain tissue, with inositol even serving as an NMR biomarker in GBM diagnostics [27,47]. However, little is known about the role of these solutes’ transport in GBM cell invasion and migration. We therefore examined in two highly motile GBM cell lines, DK-MG and SNB19, (1) the impact of elevated concentrations of glucose and inositol on the migratory activity by wound healing assays, (2) the subcellular expression patterns of SLC5A1 and SLC5A3 proteins by fluorescent immunostaining and (3) the GBM membrane permeability for glucose and inositol by osmotic swelling assay.

### 3.1. Wound-Healing and Chemotaxis Assays

**Figure 1:**
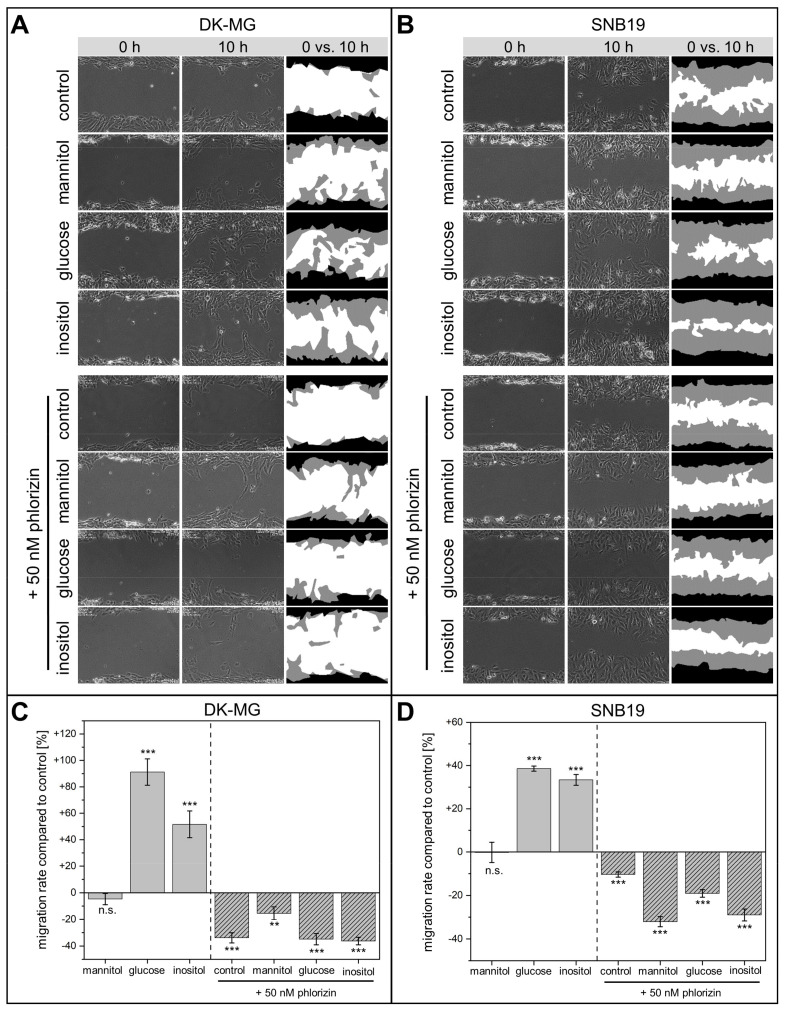
Wound healing assays of DK-MG and SNB19 cells incubated in culture medium supplemented with either mannitol, glucose or inositol or treated with phlorizin. Representative phase contrast images of wounded DK-MG (**A**) and SNB19 (**B**) cell monolayers shown in the left and middle columns were acquired at 0 and 10 h. The white color in the RHS column of A and B represents the cell-free area, and the black and gray colors denote cell-covered areas at 0 and 10 h, respectively. (**C**,**D**) Bar graphs summarizing the impact of organic solute-supplemented medium or phlorizin on the wound closure rate of DK-MG (**C**) and SNB19 cells (**D**), expressed in percentage difference to control. Each bar represents the mean ± SE of at least three independent experiments conducted in quadruplicate. “**” and “***” denote *p* <0.01 and *p* < 0.001, respectively; “n.s.” means “not significant”.

Using time-lapse video-microscopy, we first examined the wound closure rates in DK-MG and SNB19 cell monolayers exposed to slightly hypertonic growth media supplemented with 30 mM glucose or inositol. In the hypertonic control experiments, the medium was instead supplemented with 30 mM of metabolically inert mannitol (Figure 1A,B). The data for the wound healing assay on both cell lines are statistically summarized in Figure 1C,D.

We found that glucose significantly accelerated the wound closure in both DK-MG and SNB19 cell monolayers by 91% and 39%, respectively, as compared to isotonic controls. Likewise, inositol also increased the wound closure rate in DK-MG and SNB19 monolayers by 52% and 33%, respectively.

In contrast, although some studies have reported that medium tonicity impacts cell migration [17,48], we found that increasing extracellular osmolarity via metabolically inert mannitol did not affect GBM migration rates (Figure 1C,D). The increased migration speed in cells incubated in inositol- and glucose-supplemented medium thus cannot be attributed to higher medium osmolarity but stems from the specific osmolyte used as supplement.

Medium supplementation with 50 nM phlorizin, an inhibitor of both SLC5A1 [45] and SLC5A3 [46], significantly impaired the migration of both cell lines (Figure 1). Thus, phlorizin-treated DK-MG (Figure 1C) and SNB19 cells (Figure 1D) exhibited 34% and 10% decreased wound healing rates, respectively, as compared to drug-free controls. The inhibitory effect of phlorizin on the wound closure of both cell lines persisted even in the presence of supplemental solutes, including mannitol, glucose or inositol.

**Figure 2:**
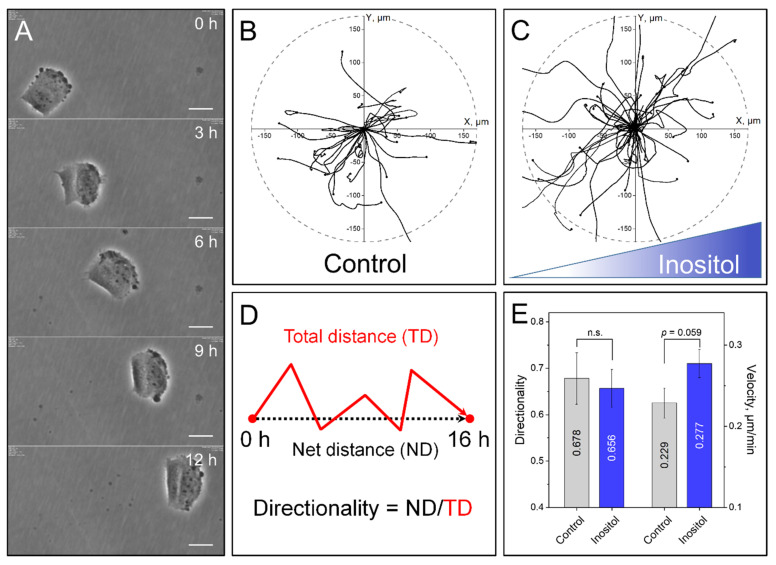
Chemotaxis experiments via µ-slide assays. (**A**) DK-MG cells were automatically tracked by the software TLA, which is able to consistently detect the cells center. Scale bar: 10 µm. (**B**,**C**) Plotting of the single tracks in a coordinate system shows directional movement, but without favoring the movement along the gradient, which increased from left to right in the *x*-axis. N_control_ = 24, N_inositol_ = 42. Endpoints were only partially marked for better visibility of tracks at the center. (**D**) Definition of directionality. (**E**) Cell directionality and total velocity were calculated. Differences in directionality were not significant, while the presence of SOOs slightly increased cell velocity (±SD). However, Student’s *t*-test showed *p*-values of >0.05, making the differences insignificant.

The faster cell migration in the presence of inositol revealed by the wound-healing assay prompted us to explore whether inositol can act as a chemoattractant. To this end, we conducted a chemotaxis assay of individual DK-MG cells (Figure 2A) in a microfluidic channel, along which an inositol gradient of 30 mM/100 µm was maintained. Because of their directionally persistent migration [5], DK-MG cells appeared most suitable for chemotaxis experiments. Single-cell migration was examined with the in-house modified software Time Lapse Analyzer, which yields individual cell trajectories (Figure 2B,C) along with data on migration speed and directionality (Figure 2E). In agreement with our previous study [5], individual DK-MG cells display a single large lamellipodium with multiple transient blebs, easily identified in phase contrast microscopy as dark spherical protrusions at the leading edge (Figure 2A).

As is evident from the tracking diagrams in Figure 2C, which show no preferential migration along the applied inositol gradient, this solute does not exert any chemotactic effect on DK-MG cells. However, consistent with the strongly polarized morphology of DK-MG cells, cell tracking (Figure 2B,C) reveals a relatively high degree of migration directionality (>0.65, Figure 2E), defined as the net distance from the starting position divided by the length of the total distance (Figure 2D). While the directionality of DK-MG cells exposed to inositol showed no significant difference from controls (Figure 2E), the final positions of a large portion of inositol-treated DK-MG cells were more distant from the starting point (Figure 2B,C, respectively). These findings, statistically summarized in Figure 2E, imply a higher migration speed of individual DK-MG cells in the presence of inositol, which is in line with the results of wound healing in DK-MG cell monolayers (Figure 1).

### 3.2. SLC5A1 and SLC5A3 Preferentially Localize to the Leading Edge of GBM Cells

**Figure 3:**
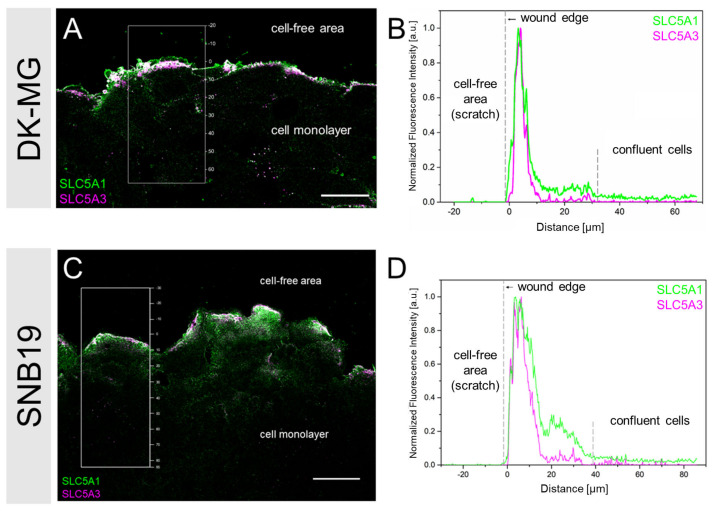
Representative LSM images of a DK-MG (**A**) and a SNB19 (**C**) cell monolayer, stained for SLC5A1 and SLC5A3 3 h after wounding. (**B**,**D**) Intensity profiles of the boxed area in A and C. Cells at the wound edge (0–~35 µm) display marked fluorescence signal peaks facing the cell-free area. In contrast, confluent cells were almost devoid of SLC5A1 and SLC5A3 signals. Scale bar in (**A**,**C**): 30 µm.

**Figure 5:**
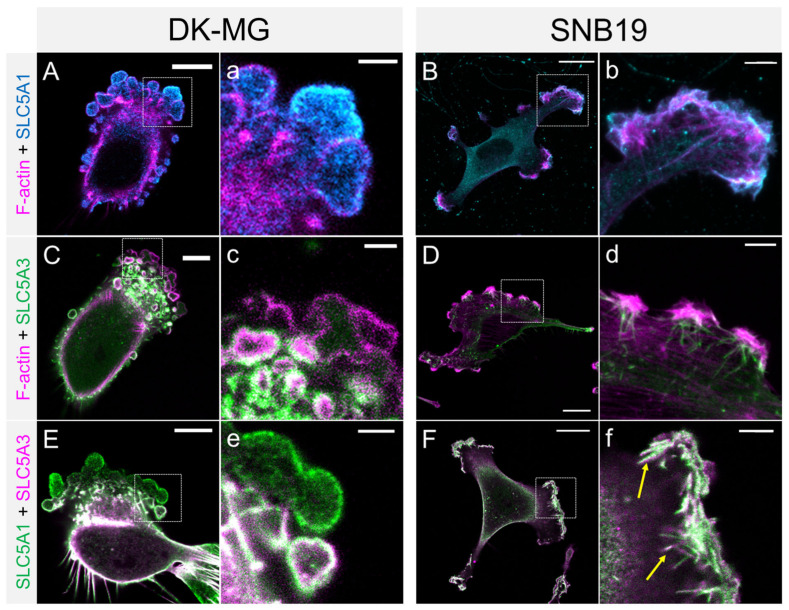
Confocal fluorescence images of DK-MG and SNB19 cells co-stained for F-actin and SLC5A1 (**A**,**B**), F-actin and SLC5A3 (**C**,**D**) or SLC5A1 and SLC5A3 (**E**,**F**). Co-localization of SLC5A1 and SLC5A3 is depicted in white. SLC5A1 predominantly localized to the cell compartments involved in migration, i.e., lamellipodia (**B**–**F**) and blebs (**A**,**C**,**E**). Lowercase letters show magnifications of the boxed regions in (**A**–**F**). Both transporter proteins display colocalization in lamellipodial tips of SNB19 cells (arrows in **f**). Scale bars: 15 µm in (**B**–**F**), 10 µm in (**A**,**C**,**E**), 5 µm in (**d**), 2.5 µm in (**a**–**c**,**e**,**f**).

Confocal laser-scanning microscopy of immuno-stained wounded DK-MG cell monolayers revealed that both transporter proteins were predominantly expressed in cells constituting the edge of the monolayer facing the wound area (Figure 3A). In contrast, cell-covered areas within the cell monolayer, consisting of non-motile cells, were virtually devoid of both transporters (bottom half of Figure 3A). This is even more evident from the intensity profiles (Figure 3B) of the boxed areas in Figure 3A, which shows that the fluorescence signals of both transporters reach their peak values at ~5 µm from the wound border. Thereafter, SLC5A1 fluorescence gradually decreases up to the distance of ~10 µm and finally falls to background level at ~35 µm, which roughly matches the lateral dimension of a single adherent DK-MG cell (see below Figure 5A). In contrast, the SLC5A3 signal already vanished ~10 µm from the wound border, indicating that expression of this transporter is confined to the leading cell edge. Qualitatively similar fluorescence distributions of both transporter proteins were found in the wounded SNB19 cell monolayers (Figure 3C,D).

Prompted by the above findings in multicellular monolayers, we further analyzed the subcellular distribution of SLC5A1 and SLC5A3 in single migrating DK-MG and SNB19 cells. In a previous study [5], we found that the two cell lines differed greatly in morphology and migration behavior. DK-MG cells maintained a unipolar morphology with a single lamellipodium, leading to directionally persistent migration. In contrast, the randomly migrating SNB19 cells displayed multiple lamellipodia.

**Figure 4:**
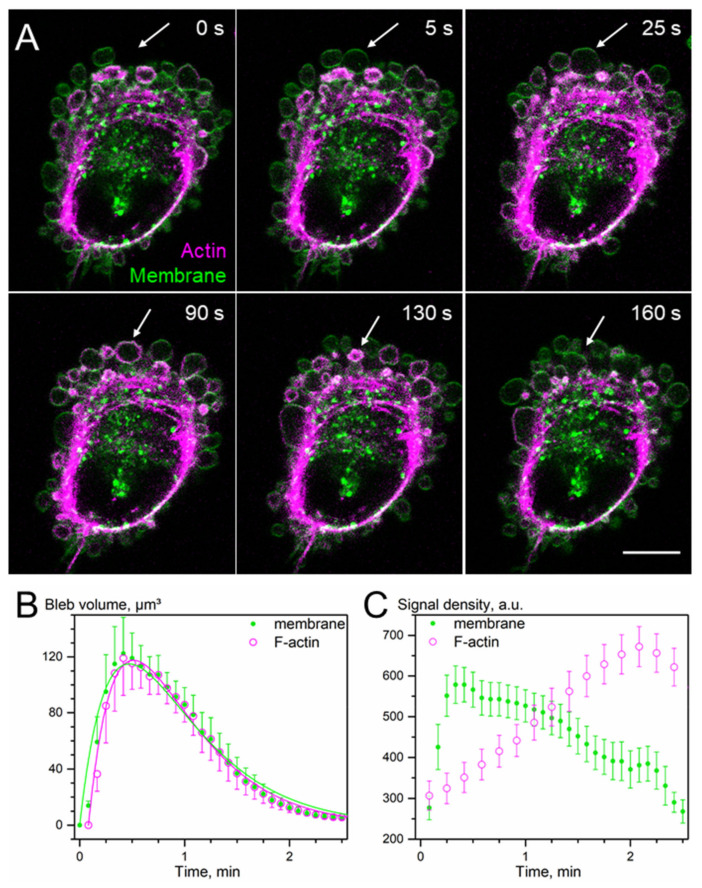
Membrane blebs in DK-MG cells. (**A**) Live-cell imaging of a representative blebbing DK-MG cell stained for F-actin (magenta) and plasma membrane (green) reveals individual bleb dynamics over time (white arrow). Scale bar: 10 µm. (**B**) Statistically summarized biphasic kinetics of membrane and F-actin formation shows a temporal shift in bleb growth. (**C**) Plotting of fluorescence intensity of plasma membrane and F-actin signals in individual blebs over time reveals a lag in F-actin signal build-up compared to the plasma membrane.

In the following single cell immunofluorescence experiments, in addition to SLC protein, we also stained actin filaments in order to visualize the morphological details of the leading cell edge, such as lamellipodia, ruffles and blebs. In particular, actin staining in DK-MG cells, exhibiting high blebbing activity at the leading edge of the lamellipodium [5], allowed us to infer the stage of the bleb life cycle from the thickness of its actin cortex [15,49]. To visualize in real time the formation of the actin cortex in the blebs of DK-MG cells, we performed dual live-cell fluorescence labeling of the cell membrane and actin (Figure 4). Judging from the membrane staining (green signal in Figure 4A), the bleb indicated by the arrow increases rapidly in size (Figure 4A, 5 s) and reaches its maximum diameter 25 s after the onset of bleb expansion. During the expansion phase (Figure 4A, time < 25 s), the bleb did not display any significant actin fluorescence. Only during the following retraction phase, i.e., between 25 and 90 s (Figure 4A), the actin cortex becomes visible and its thickness increases as bleb retraction progresses (Figure 4A, 130 s). The biphasic kinetics of bleb growth to the maximum volume of 120 fL at ~30 s and retraction during the following ~2 min are statistically (N = 8) summarized in Figure 4B, while Figure 4C shows the time courses of the actin and membrane signal densities within the bleb area. Interestingly, the kinetics of the membrane signal density (green symbols in Figure 4C) matches well the bleb volume kinetics (Figure 4B). In sharp contrast, actin signal density gradually increases during both the growth and retraction phases, which is consistent with the role of acto-myosin contraction in bleb shrinkage [49,50].

In light of the above live-cell observations (Figure 4) and data reported elsewhere [15,49], the absence of cortical actin in the fixed immunostained cells shown below signifies nascent blebs in the growth phase, whereas a thicker actin cortex indicates matured retracting blebs. We found that in DK-MG cells, SLC5A1 frequently localized to nascent expanding blebs, readily identifiable by the absence of cortical actin (Figure 5 A,a). In contrast, SLC5A3 exclusively localized to mature, retracting blebs, evident from their thick actin cortex (Figure 5C,c), whereas nascent blebs were virtually devoid of SLC5A3. This is particularly evident from the samples co-stained for both transporter proteins (Figure 5E). Interestingly, SLC5A1 and SLC5A3 co-localized only in smaller, retracting blebs, but not in the large anterior nascent blebs (Figure 5e).

In agreement with our previous study [5], single SNB19 cells displayed a multipolar morphology with numerous lamellipodia (Figure 5B,D). In addition to the diffuse fluorescence of actin-rich membrane ruffles (magenta in Figure 5B,D), the tips of lamellipodia exhibited marked expression of both transporter proteins SLC5A1 and SLC5A3 (Figure 5B,D, respectively). Co-staining of SLC5A1 with SLC5A3 reveals multiple spike-like membrane protrusions, most likely representing filopodia extending upward from the lamellipodial tip (arrows in Figure 5f). In contrast to the differential localization of SLC5A1 and SLC5A3 in DK-MG cell blebs (Figure 5e), the two transporter proteins strongly co-localized in the lamellipodial tips of SNB19 cells (Figure 5F).

Irrespective of the supplementing solute (mannitol, glucose or inositol), cultivation in slightly hypertonic (+30 mOsm) growth medium did not noticeably affect the intracellular distribution patterns of the two transporter proteins in both DK-MG and SNB19 cell lines, as seen, respectively, in Supplementary Materials Figures S2 and S3. Thus, similar to isotonic controls, hypertonically cultured DK-MG cells expressed SLC5A1 throughout the entire lamellipodium, including the membrane of the anterior-most nascent blebs, which, however, were completely devoid of SLC5A3. Interestingly, even slight hypertonicity seems to affect the morphological appearance of the leading edge of DK-MG cells, where one or a few giant blebs of irregular shape are evident (yellow arrows in Figure S2), formed, most likely, by fusion of several regular-sized blebs, as seen in isotonic cells (Figure 5 and white arrows in Figure S2).

Interestingly, LSM images of DK-MG cells exposed to 50 nM phlorizin 16 h prior to fixation displayed barely any SLC5A1 and SLC5A3 signal at the leading edge of the cell compared to untreated controls (Supplementary Materials Figure S2). Furthermore, phlorizin-treated DK-MG cells showed virtually no migratory blebs. In contrast, phlorizin-treated SNB19 cells showed both SLC5A1 and SLC5A3 signals throughout their multiple lamellipodia (Supplementary Materials Figure S3). However, phlorizin apparently impairs the formation of filopodia (Supplementary Materials Figure S3, inset).

Fluorescence staining of the microtubule protein β-tubulin, involved, among other functions, in intracellular vesicle trafficking [51,52], reveals the microtubular cytoskeleton as a pervasive cytosolic network in both DK-MG (Figure S4A,D) and SNB19 cell lines (Figure S4G,J). Interestingly, SLC5A1 strongly co-localizes with tubulin in large nascent blebs of DK-MG cells (yellow arrows, Figure S4B), whereas SLC5A3, confined to mature blebs, displays no such co-localization with tubulin (Figure S4D–F). In SNB19 cells, microtubules extend up to the lamellipodial tips, where they co-localize with both SLC5A1 (Figure S4G–I) and SLC5A3 (Figure S4J–L).

### 3.3. Super-Resolved (dSTORM) Images of SLC5A1, SLC5A3, F-Actin and Tubulin

**Figure 6:**
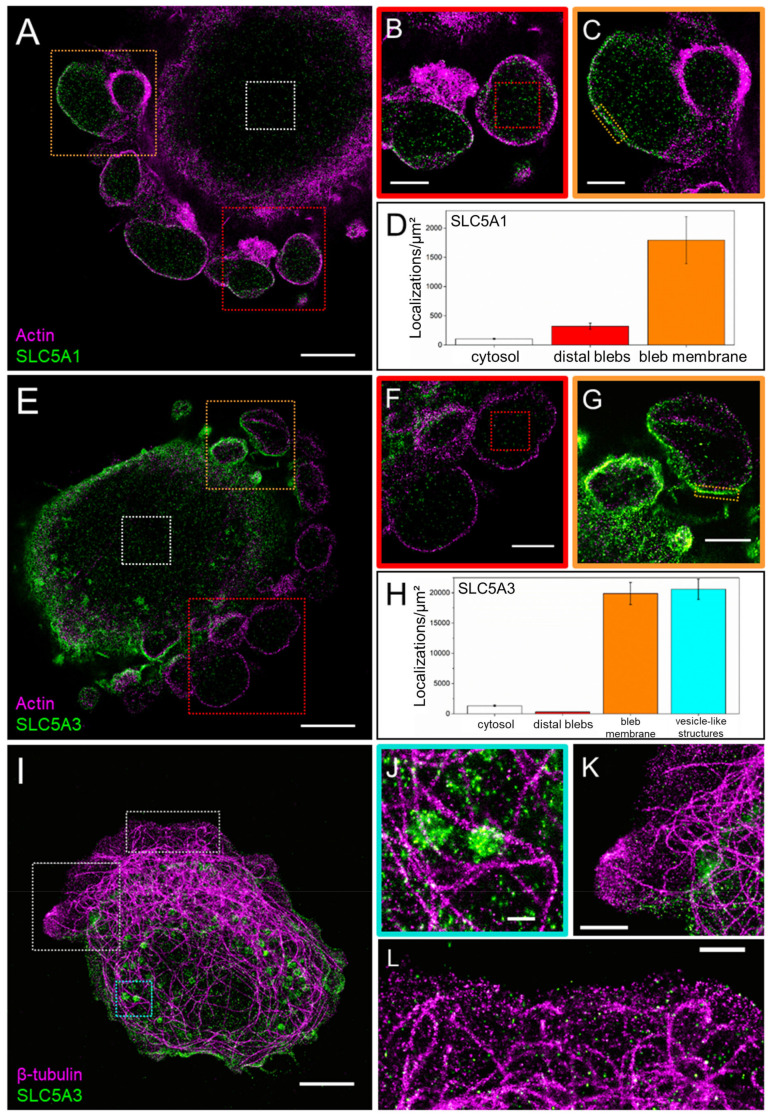
Two-color *d*STORM analyses of SLC5A1 and SLC5A3 in DK-MG cells. (**A**) Representative *d*STORM image of a DK-MG cell co-stained for SLC5A1 and F-actin. (**B**,**C**) Magnifications of the boxed regions in (**A**), depicting blebs with (**B**) or lacking (**C**) an actin cortex. (**D**) SLC5A1-localizations per µm^2^ in different cell compartments corresponding to the boxed regions in (**A**). The highest localization densities were found in the membrane of blebs most distant from the main cell body. (**E**) Representative *d*STORM image of a DK-MG cell co-stained for SLC5A3 and F-actin. (**F**,**G**) Magnifications of the boxed regions in (**E**), depicting distal blebs lacking SLC5A3 signal (**F**) and blebs in various life cycle stages (**G**). (**H**) SLC5A3-localizations per µm^2^ in different cell compartments corresponding to the boxed regions in (**E**). The highest localization densities were found in the membrane of blebs closest to the main cell body (**F**), as well as in vesicle-like structures close to the cell periphery (**J**). (**I**) Representative *d*STORM image of a DK-MG cell co-stained for SLC5A3 and β-tubulin. (**J**) Vesicle-like SLC5A3 structures localized in close vicinity to microtubules. (**K**,**L**) Microtubules extending into the outermost leading edge, virtually devoid of SLC5A3. Scale bars: 5 µm in (**A**,**E**,**I**); 2 µm in (**B**,**C**,**F**,**G**), 1 µm in (**K**,**L**); 500 nm in (**J**).

In addition to conventional microscopy, we employed super-resolution microscopy to visualize, with molecular resolution, the localization of both transporter proteins in blebbing DK-MG cells along with actin and tubulin cytoskeleton, as shown in Figure 6. In agreement with our CLSM data (Figure 5), DK-MG cells display by far the highest localization density of SLC5A1 (~1.8 × 10^3^ loc/µm^2^) in the membrane of large expanding blebs devoid of actin cortex (Figure 6C,D). For comparison, the bulk cytosol and the bleb interior exhibit much lower SLC5A1 densities of ~10^2^ and ~3 × 10^2^ loc/µm^2^, respectively (Figure 6D).

Unlike SLC5A1, SLC5A3 shows its highest localization density in the membrane of small, retracting blebs (Figure 6G; ~2.0 × 10^4^ loc/µm^2^), exceeding by 1–2 orders of magnitude the density of SLC5A3 protein reported for the basal membrane of osmotically stressed HEK293 cells [19]. In contrast, blebs in the early stage of retraction, discernable by their newly formed thin actin cortex, exhibit barely any SLC5A3 signal (~3 × 10^2^ loc/µm^2^, Figure 6E,F). These results suggest that SLC5A3 expression in the bleb membrane increases during bleb retraction.

Although the bulk cytosol exhibits only sparse localization of SLC5A3 (~1.3 × 10^3^ loc/µm^2^), numerous spherical structures in the cytosol with a diameter of ~0.5 µm (Figure 6I) contain large amounts of SLC5A3 (Figure 6H). The very high SLC5A3 density of ~2.3 × 10^4^ loc/µm^2^ found in the cytosolic spherical structures is strikingly similar to that observed in the membrane of mature retracted blebs (Figure 6H), which suggests that these SLC5A3-rich structures represent endocytic vesicles involved in SLC5A3 recycling. This notion is further reinforced by the close localization of SLC5A3 vesicles to microtubules (Figure 6J), a well-known component of vesicle trafficking [51,52]. Finally, in agreement with our LSM images (Supplementary Materials Figures S2 and S3), *d*STORM revealed a dense intertwined meshwork of microtubules, most notable in the lamellae of DK-MG cells, with individual microtubules even extending to the anterior-most blebs. The pronounced microtubular network is indicative of high vesicular trafficking activity in the lamellipodium, which is also evident from phase-contrast live-cell imaging (Supplementary Materials Video S1).

### 3.4. Osmotic Swelling Assay for Glucose and Inositol

The high expression of both transporter proteins SLC5A1 and SLC5A3 in the membranes of GBM cells (Figure 5) is likely associated with substantial membrane permeability to the respective transporter substrates. To probe the solute permeability of the cell membrane, we performed osmotic cell swelling experiments in hypotonic solutions containing either glucose, inositol, mannitol or sucrose as the major solute.

Cell volume changes were monitored by video microscopy following the rapid transfer of cells from isotonic CGM (~300 mOsm) to a hypotonic sugar solution. Figure S5 shows the mean volumetric response of the two cell lines to various sugar solutions with the same osmolality of 150 mOsm. Independent of the sugar used, the sudden exposure to hypotonicity caused both GBM cell lines to swell rapidly within the first 3–5 min from their original isotonic volume V_0_ to the V_max_ level due to the fast water influx driven by the applied osmotic gradient. Hypotonic solutions of all tested sugars gave rise to qualitatively similar rates and magnitudes (~20%) of initial swelling in both cell lines.

The data in Figure S5A,B reveal striking differences between the disaccharide sucrose and the monomeric sugars and sugar-alcohols, including glucose, inositol and mannitol, in respect to their effects on the secondary cell volume changes. After the fast initial swelling in hypotonic sucrose solution, both cell lines underwent a regulatory volume decrease (RVD), during which they shrank gradually, fully recovering their original isotonic volume (V_0_) within ~15–20 min despite persisting hypotonicity. RVD relies on the release of cytosolic solutes through swelling-activated membrane pathways accompanied by osmotically obligated water efflux, which allows cells to recover their original isotonic volume [19,53]. In agreement with our findings for DK-MG and SNB19 cells presented here (Figure S5) and elsewhere [54], other cell lines, including glioma cells, are able to quickly readjust their volume in anisotonic media via several mechanisms, including chloride pathways and aquaporins [55,56,57], only in the presence of membrane impermeable solutes, such as dimeric sugars, sucrose or trehalose [39].

In sharp contrast to the disaccharide sucrose, the monomeric sugars partially (mannitol) or completely abolished the RVD (glucose and inositol) in DK-MG cells (Figure S5A). In SNB19 cells, glucose, mannitol and inositol not only abolished RVD, but even induced secondary swelling (Figure S5B). As shown elsewhere [58], RVD inhibition is based on the uptake of extracellular monomeric sugars into the cytosol through swelling-activated membrane pathways. The influx of permeable extracellular solutes compensates for the loss of cytosolic osmolytes, thus preventing RVD. As expressed by Equation (1) (see Materials and Methods), the extent of RVD inhibition by various solutes is indicative of their membrane permeabilities [40]. Accordingly, an *IC_RVD_* ≈ 0 means complete recovery of initial cell volume by RVD, i.e., in the presence of the impermeable sucrose. The range 0 < *IC_RVD_* < 100% indicates partial-to-complete RVD inhibition. Highly permeable solutes, such as glucose, mannitol and inositol in SNB19 cells, cause secondary cell swelling and thus exhibit *IC_RVD_* >> 100% (200–222%).

The values of *IC_RVD_* [%] calculated with Equation (1) from the data in Figure S5A yield the following descending rank order of solute permeability in DK-MG cells: *inositol* (90%) ≈ *glucose* (78%) > *mannitol* (41%) > *sucrose* (0%). In contrast to DK-MG cells, glucose displayed by far the highest permeability among the tested solutes in SNB19 cells: *glucose* (222%) > *inositol* (209%) > *mannitol* (~200%) >> *sucrose* (~0%). Taken together, the data in Figure S5 suggest that even a slight cell volume increase of ~25% rendered the GBM cell membranes highly permeable to glucose and inositol. Similar to the hypotonic activation of SLC5A3 in HEK293 cells [19], swelling-activated pathways for glucose and inositol in GBM cells reported here might also include SLC5A1 and SLC5A3 transporters.

## 4. Discussion

Our findings, briefly summarized below, suggest that SLC5A1 and SLC5A3 are involved in GBM cell migration. Medium supplementation with glucose and inositol increased migration rates in both cell lines by 33–91% above the respective controls (Figure 1C,D). The significant increase in wound healing rate displayed by DK-MG and SNB19 cells (+52% and +33%, respectively) in the presence of inositol (Figure 1C,D) is likely connected to inositol serving as a precursor of phosphatidylinositol 4,5-bisphosphate (PIP2), a key signaling lipid in cancer cell migration [38]. The observed increased migration rate of GBM cells exposed to glucose (+91% in DK-MG cells; +39% in SNB19 cells) can be explained by the metabolic promotion of invasive cancer cell behavior by this solute. Indeed, elevated glucose levels are known to promote cancer cell migration [59,60,61]. In contrast, the SLC inhibitor phlorizin [45,46] decreased migration rates by 10–36% (Figure 1C,D). As shown in Supplementary Materials Figures S2 and S3, phlorizin altered the expression of SLC5A1 and SLC5A3 at the leading edge of both tested cell lines compared to the respective untreated controls. In agreement with our findings, Gao et al. (2019) [62] also reported that phlorizin decreased the expression of SLC proteins in cancer cells. Interestingly, the highly invasive [6] SNB19 cells exposed only to phlorizin displayed the lowest reduction in motility (−10%) of all experimental conditions involving inhibition. This corroborates our previous finding that SNB19 cells react only moderately to pharmacological inhibition of migration [5,7]. Our immunostaining of wounded GBM cell monolayers (Figure 3A,C) revealed that both SLC5A1 and SLC5A3 are highly expressed only in migrating cells at the monolayer edge but not in the non-motile cells within the confluent monolayer (Figure 3B,D). Interestingly, a variety of studies have demonstrated that such extensive remodeling of membrane solute channel expression, as seen in edge cells, is also observed during epithelial-mesenchymal transition (EMT) [63], a crucial factor in cancer cell metastasis and wound healing [64,65].

Our immuno-staining experiments on single, actively migrating DK-MG and SNB19 cells lend further support to the involvement of SLC transporters in GBM cell migration. As seen in Figure 5 and Figures S2–S4, both SLC5A1 and SLC5A3 are predominantly expressed in the lamellipodia (Figure 5B,D,F) and blebs (Figure 5A,C,E), i.e., the expanding cell protrusions known for driving cell migration [18,50,66].

Interestingly, lamellipodia expansion by local volume increase can be achieved by the import of solutes and osmotically obliged water influx [66]. Furthermore, SLC5A1 has been shown to be involved in glucose-driven modulation of membrane protrusions [67]. As we have shown in Supplementary Materials Figure S5, both DK-MG and SNB19 cell membranes are highly permeable to glucose and inositol. The presence of SLC5A1 and SCL5A3 in the lamellipodial tips of SNB19 cells (Figure 5b,f) thus points to the possible involvement of these transporters in the local volume increase required for lamellipodium protrusion during cell migration.

As pointed out elsewhere [18], cell migration requires the concerted activity of various channels and transporters belonging to diverse protein families. Efficient cell migration thus necessitates not the isolated function of a single channel or transporter but the interdependent activity of a transport protein network.

The involvement of aquaporins in bleb formation and blebbing activity [68,69,70] suggests that water flux through the bleb membrane may also play a key role in migratory cell blebbing. Since SLC5A1 was detected in nascent blebs devoid of actin cortex (Figure 5a), we can infer that this glucose transporter present in the bleb membrane during bleb expansion enabled the uptake of extracellular glucose, leading to the osmotically obligated water influx (facilitated by aquaporins), i.e., local volume increase. The high SLC5A1 levels in nascent blebs were confirmed by our nanoscale imaging (Figure 6A), in which only expanding blebs displayed a marked SLC5A1 signal (Figure 6C). Additionally, the cytosol in expanding blebs displayed significantly higher amounts of SLC5A1 compared to the cytosol of the main cell body (Figure 6D), possibly due to the dissociation of SLC5A1 from the bleb membrane.

In contrast to SLC5A1, the inositol transporter SLC5A3 was found only in smaller *retracting* blebs with a reestablished actin cortex (Figure 5c,e and Figure 6G), while the anterior-most blebs were virtually devoid of the SLC5A3 signal (Figure 6F). We therefore conclude that SLC5A3 can contribute to bleb retraction via an osmotic bleb volume decrease due to inositol/water efflux. Consistent with this assumption, SLC5A3 was previously shown to be involved in the regulatory volume decrease in HEK293 cells [19]. This notion is further supported by our finding that the SLC5A3 signal increased with increasing bleb maturity (Figure 6G). In larger blebs (upper right in Figure 6G), SLC5A3 was detected only at the bleb membrane hemisphere facing the cell body, if at all (Figure 6F).

Previous studies have demonstrated that SLCs are inserted into the plasma membrane from cytosolic vesicles during swelling-activated exocytosis [19,71]. Interestingly, our super-resolution *d*STORM imaging revealed sub-µm-sized vesicle-like clusters of SLC5A3 located in close proximity to microtubules (Figure 6I,J), suggesting that SLC protein trafficking occurs via vesicles moving along the microtubular network. Indeed, co-localization of tubulin and the taurine transporter SLC6A6 was already reported elsewhere [72]. Furthermore, SLC6A12, a transporter of SOO betaine, was found to relocate from the cytosol to the plasma membrane upon hypertonic stimulation [73]. This points to the membrane incorporation of SLC transporters via exocytosis, i.e., vesicular fusion, as a common phenomenon for proteins of the SLC family, as was also demonstrated for various other SLCs [74,75]. Accordingly, we frequently observed that in large blebs, SLC5A3 appeared at the bleb side facing the main cell body (Figure 6G, upper right corner), consistent with insertion via vesicular fusion.

Furthermore, our finding that microtubule structures extend into the anterior-most blebs virtually devoid of SLC5A3 (Figure 6K,L and Figure S4E) suggests that other SLC transporters might also be incorporated into blebs via vesicle transport along microtubules (Figure S4B). Thus, our findings provide an additional rationale for targeting microtubules in the context of inhibiting cell migration and invasion [76,77,78].

## 5. Conclusions

Taken together, the high expression of SLC5A1 and SLC5A3 transporters in the lamellipodia of GBM cells and the impaired cell motility upon SLC inhibition point to the transporters’ involvement in GBM cell migration. We thus propose to include SLC5A1 and SLC5A3 in the migration-associated transportome. The revealed SLC-related cell motility might offer therapeutic potential in GBM treatment, especially given the abundance of inositol and glucose in the human brain.

## Data Availability

Not applicable.

## Supplementary Materials

The following supporting information can be downloaded at: https://www.mdpi.com/2072-6694/14/23/5794/s1, Figure S1: Kaplan-Meier plots of overall survival and expression of SLC5A1 and SLC5A3 in GBM patients; Figure S2: Confocal images of SLC5A1/A3 in DK-MG cells treated with mannitol, glucose or inositol; Figure S3: Confocal images of SLC5A1/A3 in SNB19 cells treated with mannitol, glucose or inositol; Figure S4 Confocal images of β-tubulin, SLC5A1 and SLC5A3 in DK-MG and SNB19 cells; Figure S5: Volumetric measurements of DK-MG and SNB19 cells treated with sucrose, mannitol, glucose or inositol; Video S1: Vesicle trafficking in the lamellipodium of GBM cells.
