# Correction: Brosch et al. Glucose and Inositol Transporters, SLC5A1 and SLC5A3, in Glioblastoma Cell Migration. *Cancers* 2022, *14*, 5794

**DOI:** 10.3390/cancers15215139

**Published:** 2023-10-25

**Authors:** Philippa K. Brosch, Tessa Korsa, Danush Taban, Patrick Eiring, Philipp Kreisz, Sascha Hildebrand, Julia Neubauer, Heiko Zimmermann, Markus Sauer, Ryo Shirakashi, Cholpon S. Djuzenova, Dmitri Sisario, Vladimir L. Sukhorukov

**Affiliations:** 1 Department of Biotechnology & Biophysics, Biocenter, University of Würzburg, 97074 Würzburg, Germany; philippa.brosch@uni-wuerzburg.de (P.K.B.); tessa.korsa@ibmt.fraunhofer.de (T.K.); danush.taban@uni-wuerzburg.de (D.T.); patrick.eiring@uni-wuerzburg.de (P.E.); sascha.hildebrand@stud-mail.uni-wuerzburg.de (S.H.); m.sauer@uni-wuerzburg.de (M.S.); 2 Fraunhofer Institute for Biomedical Engineering (IBMT), 66280 Sulzbach, Germany; julia.neubauer@ibmt.fraunhofer.de (J.N.); heiko.zimmermann@ibmt.fraunhofer.de (H.Z.); 3 Julius-von-Sachs Institute, University of Würzburg, 97082 Würzburg, Germany; philipp.kreisz@uni-wuerzburg.de; 4 Department of Molecular and Cellular Biotechnology, Saarland University, 66123 Saarbrücken, Germany; 5 Faculty of Marine Science, Universidad Católica del Norte, Coquimbo 1281, Chile; 6 Institute of Industrial Science, The University of Tokyo, Tokyo 153-8505, Japan; 7 Department of Radiation Oncology, University Hospital of Würzburg, 97080 Würzburg, Germany; djuzenova_t@ukw.de

## Addition of an Author

Philipp Kreisz was not included as an author in the original publication [1]. The corrected Author Contributions Statement appears here. The authors apologize for any inconvenience caused, and state that the scientific conclusions are unaffected. This correction was approved by the Academic Editor. The original publication has also been updated.
