# Supplementary material for: Glucose and Inositol Transporters, SLC5A1 and SLC5A3, in Glioblastoma Cell Migration
Source: Cancers (Basel). 2022 Nov 24;14(23):5794. doi: 10.3390/cancers14235794 (PMC9738886; doi:10.3390/cancers14235794)
Supplement: Supplementary file 1 [file cancers-14-05794-s001.zip › Supplement Brosch et al 2022.pdf]

Supplemental figures

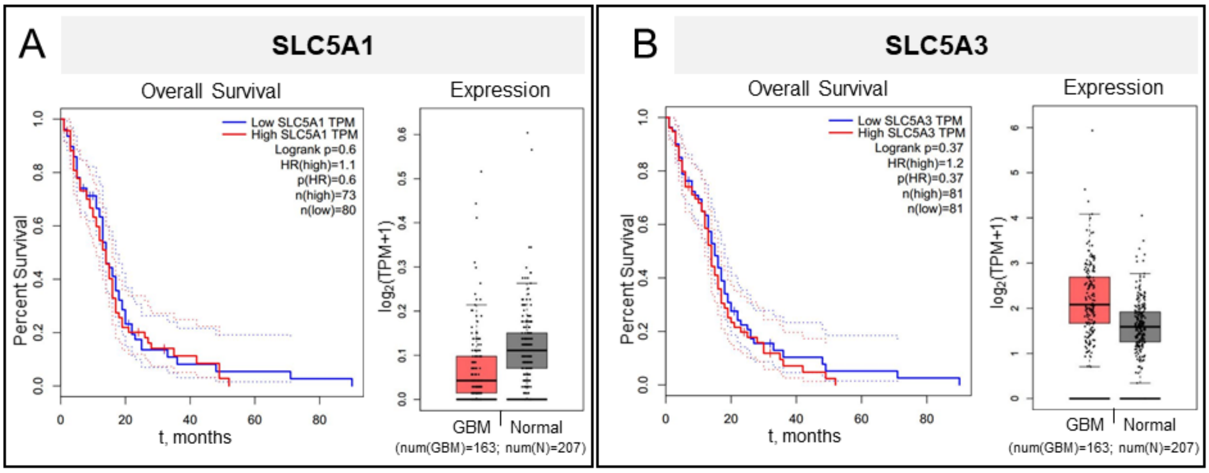

Figure S1: Kaplan-Meier plots of overall survival in GBM patients with low and high SLC5A1 (**A**, LHS) or SLC5A3 (**B**, LHS) as well as comparison of SLC5A1- (**A**, RHS) and SLC5A3-expression (**B**, RHS) between GBM patients and normal tissue.

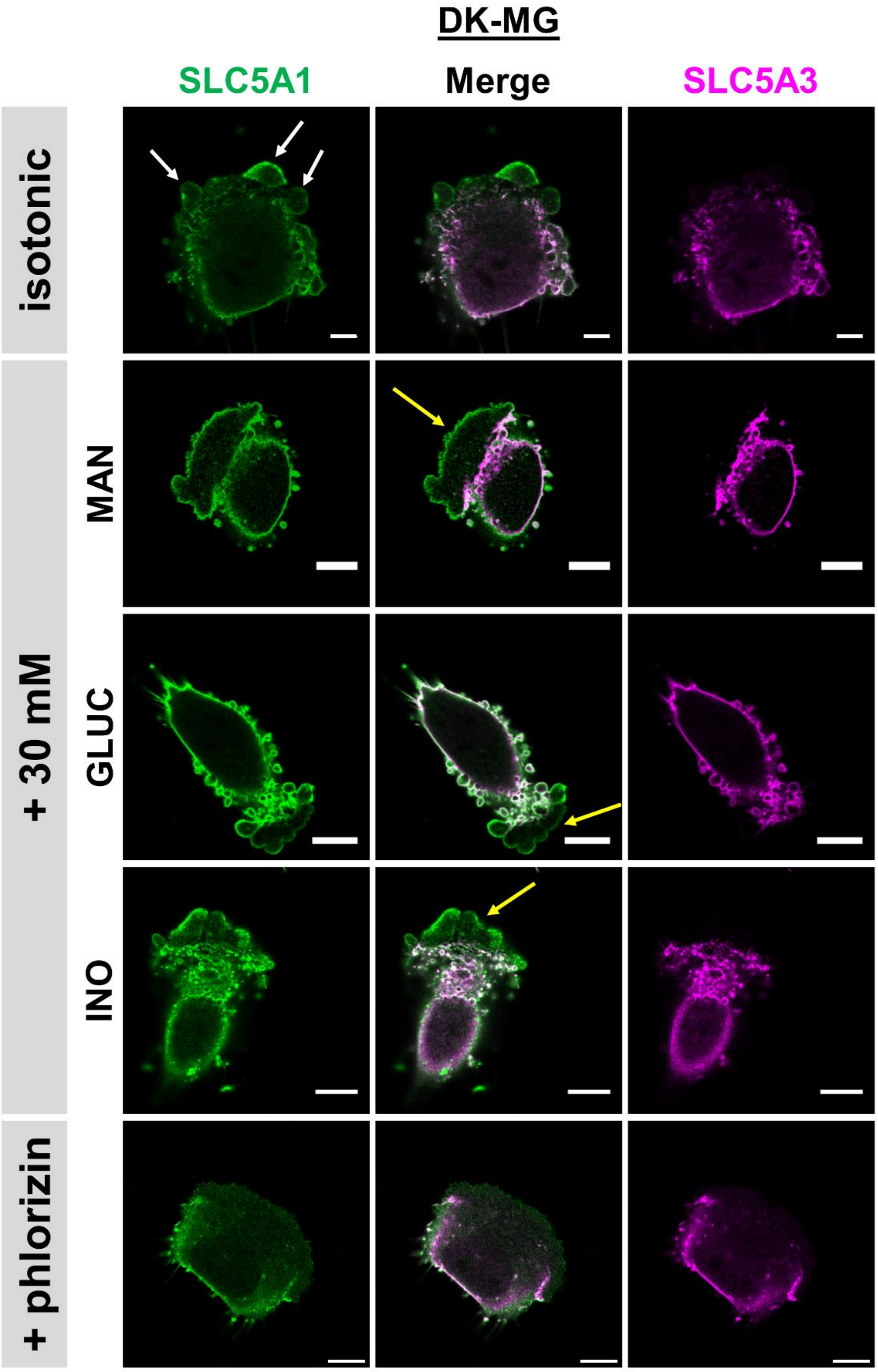

Figure S2: Representative LSM images of DK-MG cells incubated for 16 h in CGM supplemented with either 30 mannitol, glucose or inositol or exposed to 50 nM phlorizin prior to immunostaining for SLC5A1 (green) and SLC5A3 (magenta). In DK-MG cells SLC5A3 consistently did not localize to the anteriormost blebs at the front of the leading edge. Cell morphology and subcellular localization of both SLC transporters remained unchanged by the presence of hypertonic SOO solutions, except for the appearance of merged blebs (yellow arrows). Phlorizin-treated cells not only display a distinct lack of SLC5A1 and SLC5A3 at the leading cell edge, but also a virtually total disappearance of migratory cell blebs. Scale bars: 10 µm.

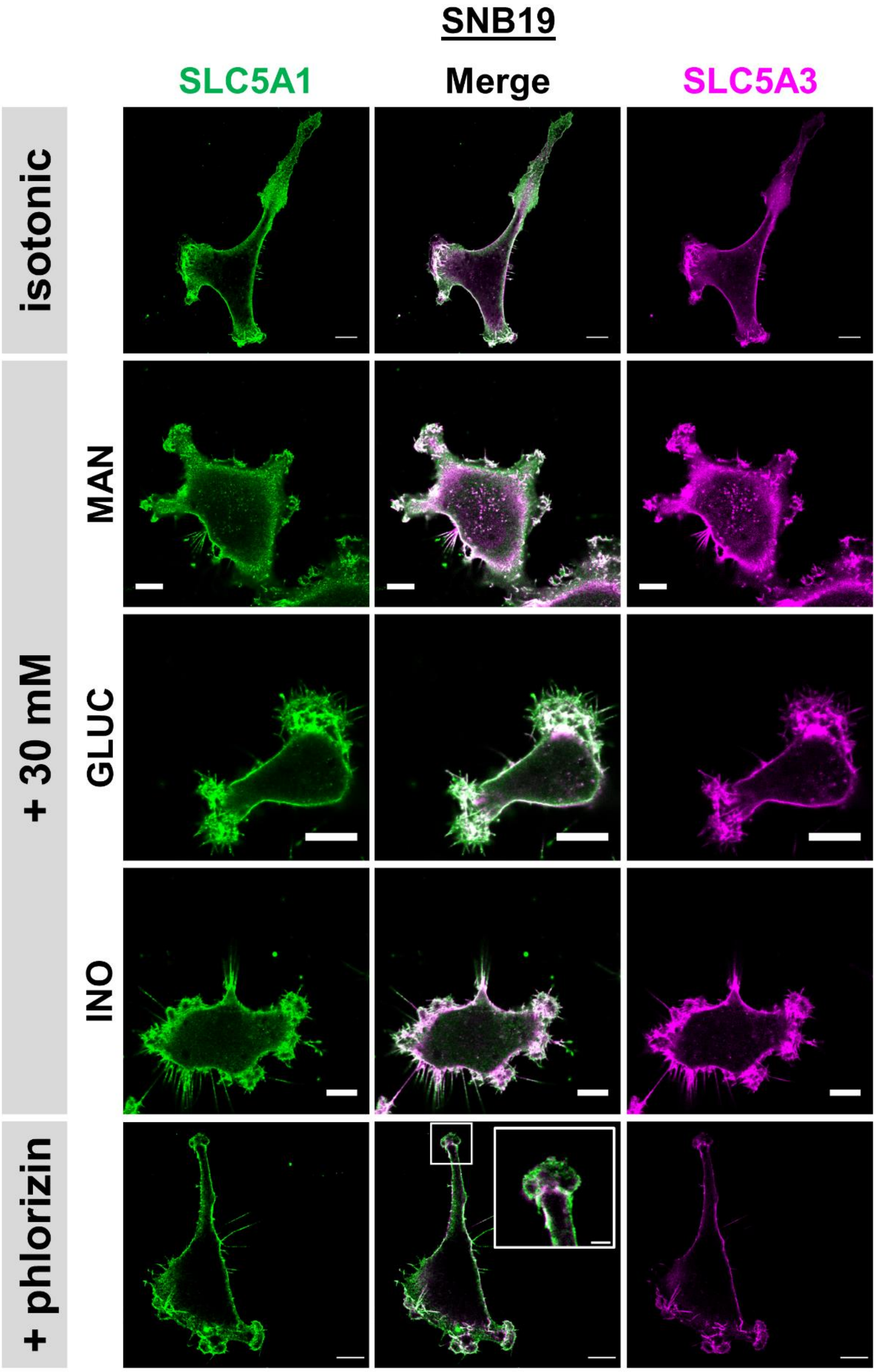

Figure S3: Representative images of SNB19 cells incubated for 16 h in CGM supplemented with either 30 mannitol, glucose or inositol or exposed to 50 nM phlorizin prior to immunostaining for SLC5A1 (green) and SLC5A3 (magenta). In SNB19 cells, no morphological differences to control cells were apparent, and the subcellular localization of both SLC transporters remained unaffected by the presence of hypertonic SOO solutions. However, the multiple lamellipodia of phlorizin-treated SNB19 cells display a lack of filopodia compared to untreated controls. Scale bars: 10 µm.

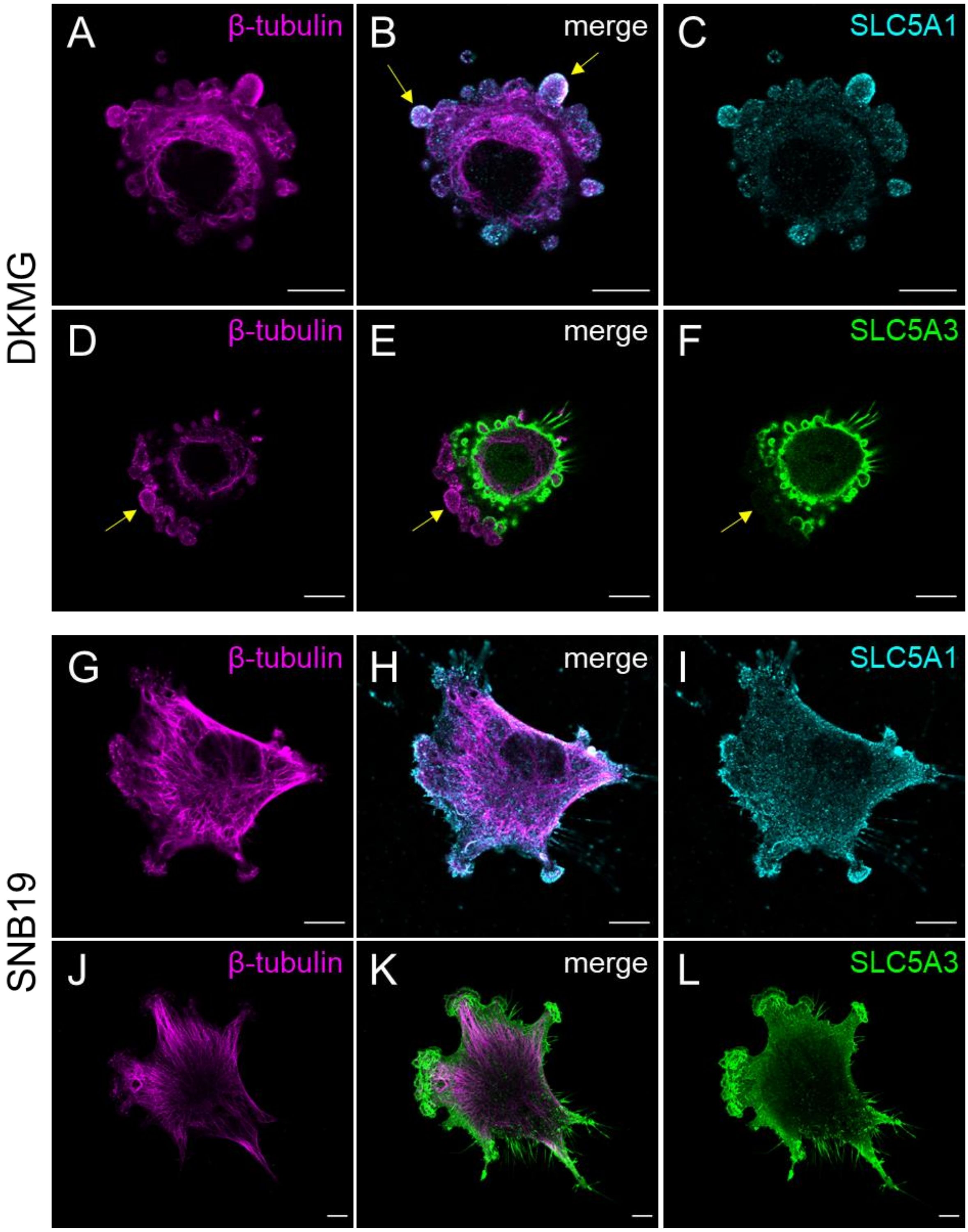

Figure S4: Laser-scanning microscopy images of GBM cells stained for β-tubulin (magenta) and either SLC5A1 (cyan) or SLC5A3 (green). **A-C**) Images of a representative DK-MG cell stained for β-tubulin (A) and SLC5A1 (C). SLC5A1 displayed distinct co-localization with β-tubulin in distal DK-MG cell blebs (arrows, B). **D-F**) Images of a representative DK-MG cell stained for β-tubulin (D) and SLC5A3 (F). SLC5A3 was only present in smaller, presumably retracting blebs (arrow, E), while the anteriormost blebs displayed no SLC5A3 signal. **G-L**) Images of a representative SNB19 cell stained for β-tubulin (G) and SLC5A1 (H, I) or SLC5A3 (K, L). The cytoskeleton protein β-tubulin extended throughout the multiple SLC5A1- and SLC5A3-rich lamellipodia. Scale bars: 10 µm.

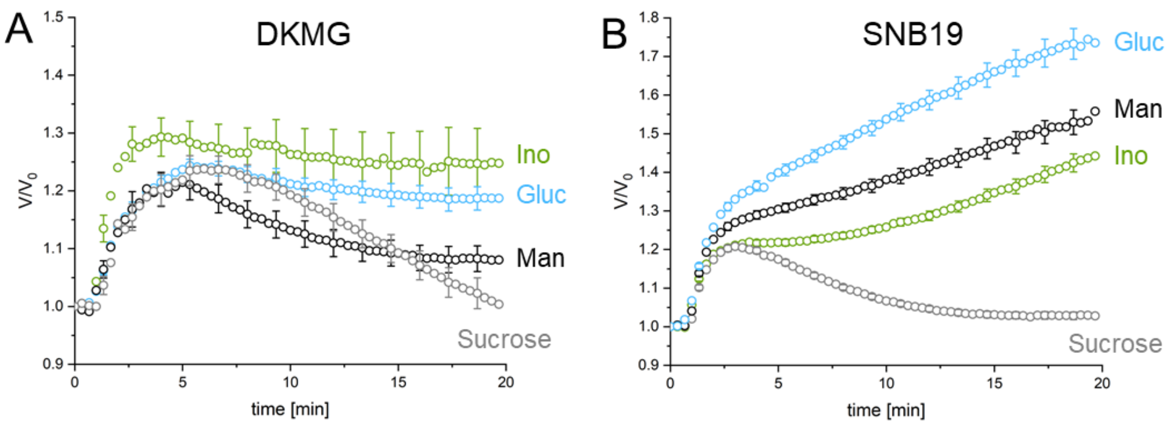

Figure S5: Impact of hypotonic solutions containing either mannitol, glucose, inositol or sucrose as the major solute on the swelling-activated permeability of DK-MG (**A**) and SNB19 (**B**) cells. Cells exposed to inositol- and glucose-solutions displayed no regulatory volume decrease.

Increased expression levels of SLC5A1 and SLC5A3 are detrimental to GBM patient survival

Using the gene expression profiling tool GEPIA2, we examined the clinical impact of the SLC5A1 and SLC5A3 gene expression on GBM progression. The line plots in Figs. S1A and S1B show the GBM patient survival for low (blue line) and high (red line) mRNA expression levels of the proteins SLC5A1 and SLC5A3 are depicted as Kaplan-Meier plots. We found that GBM patients with high SLC5A1- or SLC5A3-expression had slightly higher mortality rates, as evident from the respective hazard ratio (HR_{SLC5A1}(high) = 1.1; HR_{SLC5A3}(high) = 1.2), which compares patients with low and high expression of SLC5A1 (Fig. S1A) or SLC5A3 (Fig. S1B). This indicates that high SLC5A1 or SLC5A3 expression is detrimental to GBM patient survival.

Furthermore, comparison of the expression of SLC5A1 in normal and GBM cells (box plot in Fig. S1A) reveals that GBM expression of SLC5A1 is lower compared to healthy cells. In contrast, SLC5A3 expression was elevated in GBM cells compared to normal cells (box plot in Fig. S1B).
